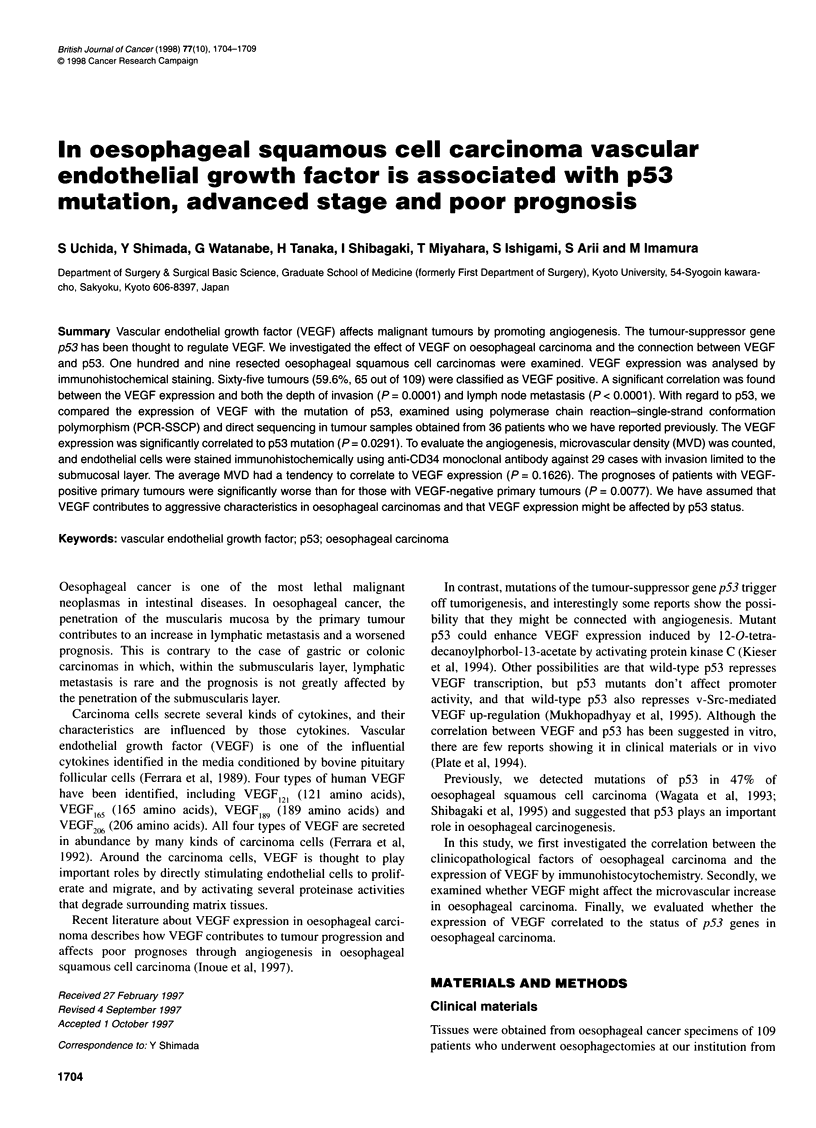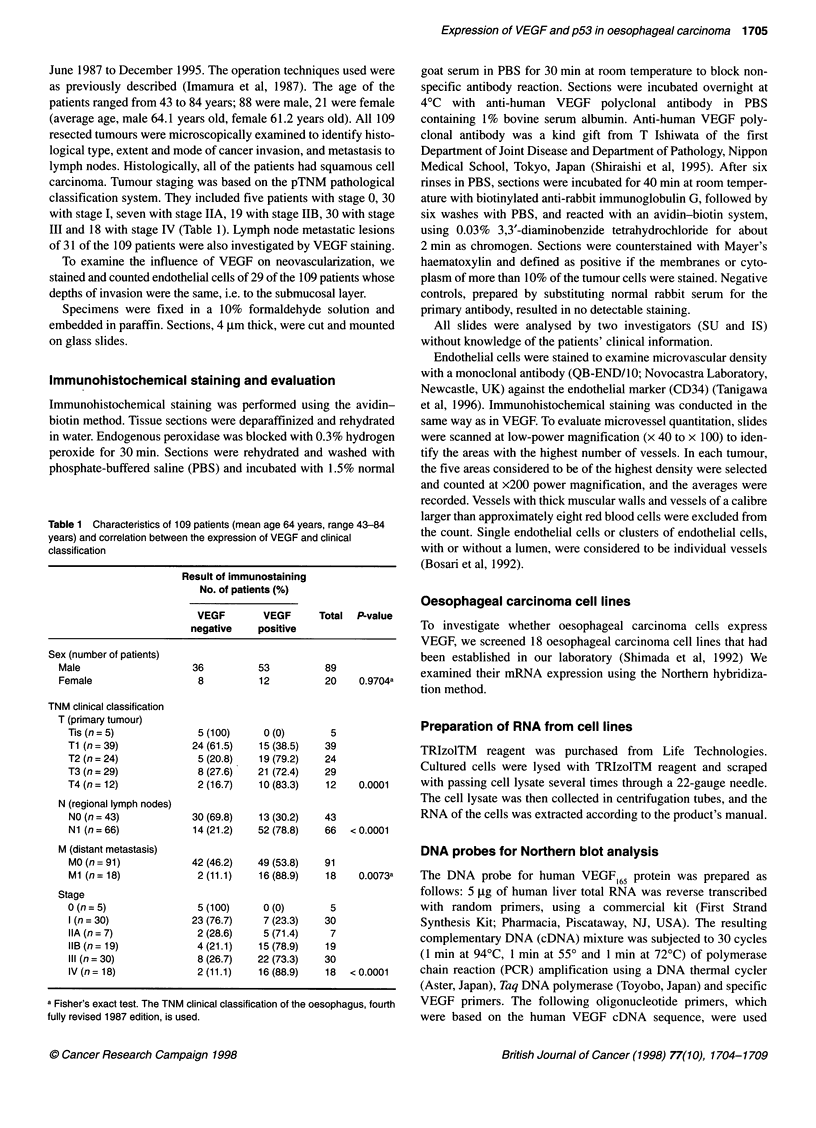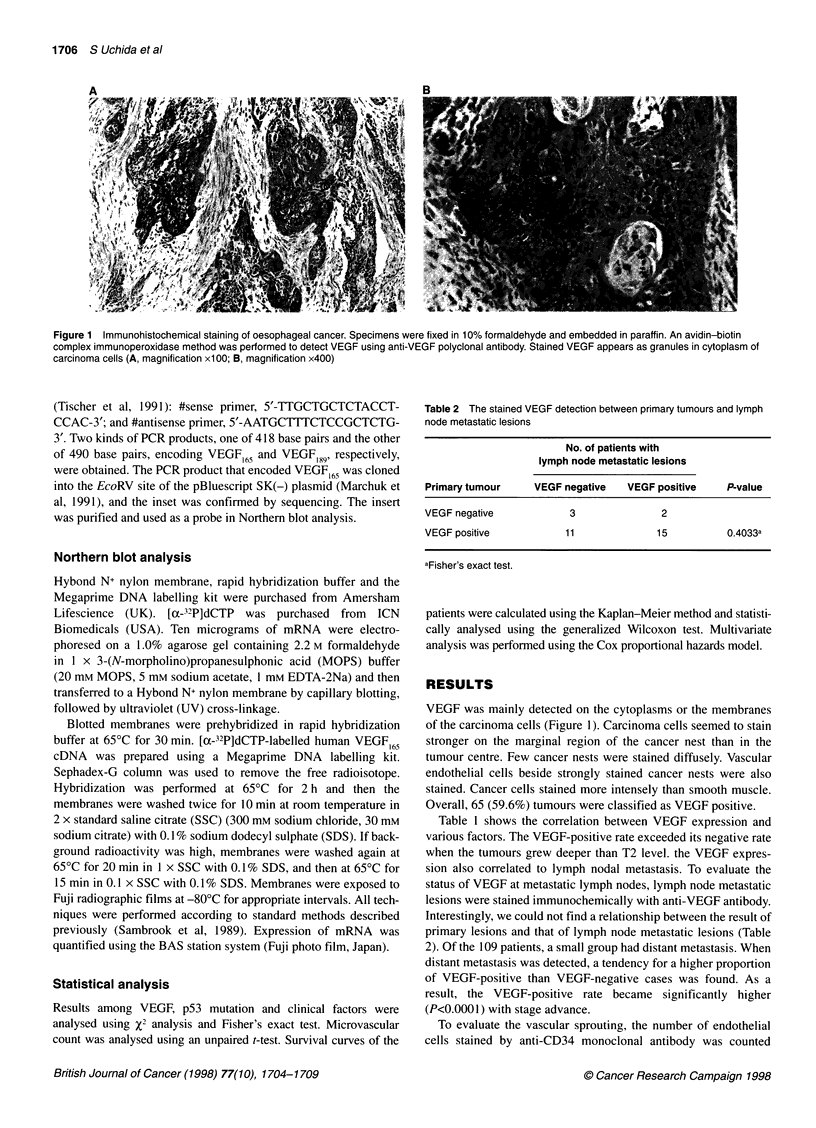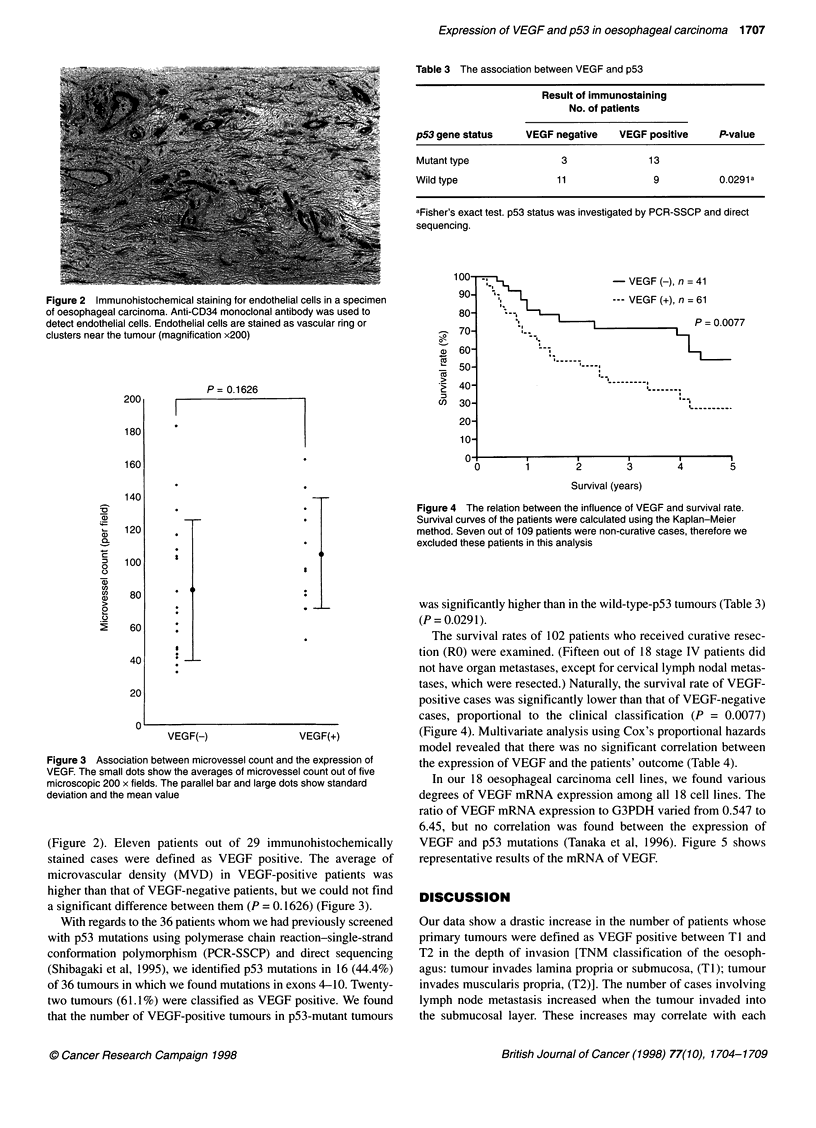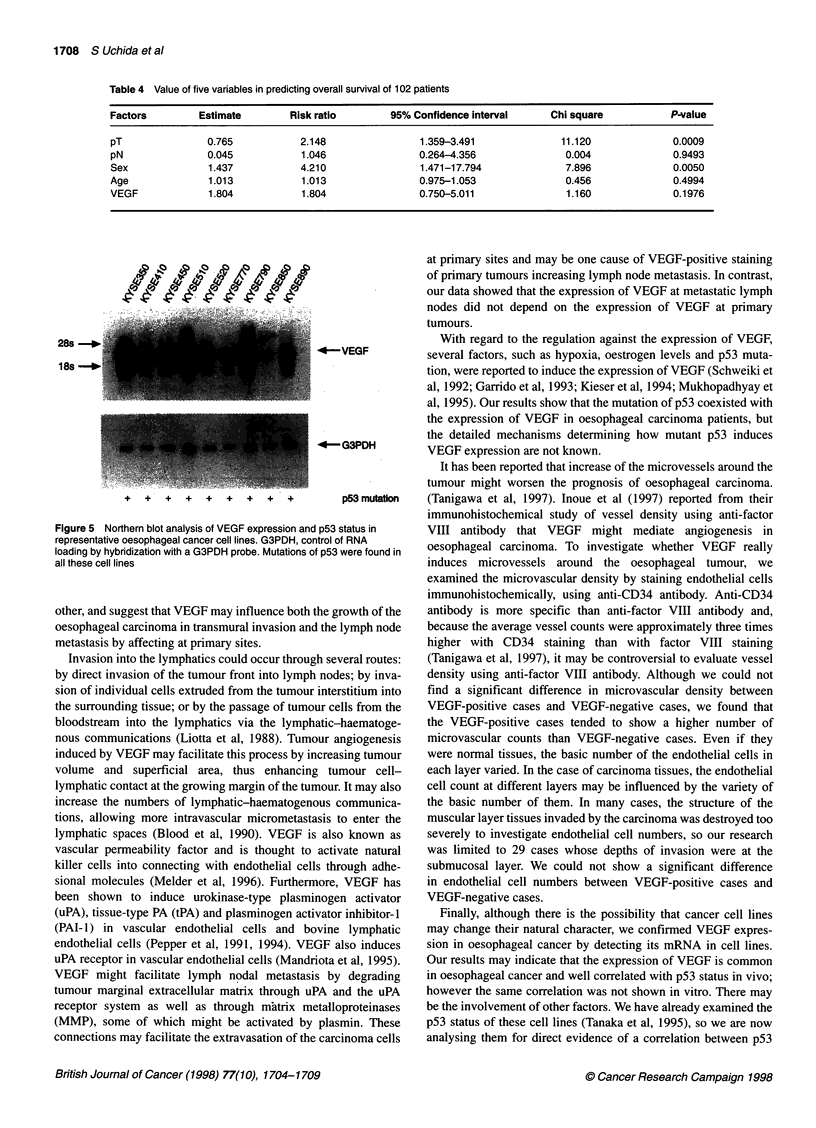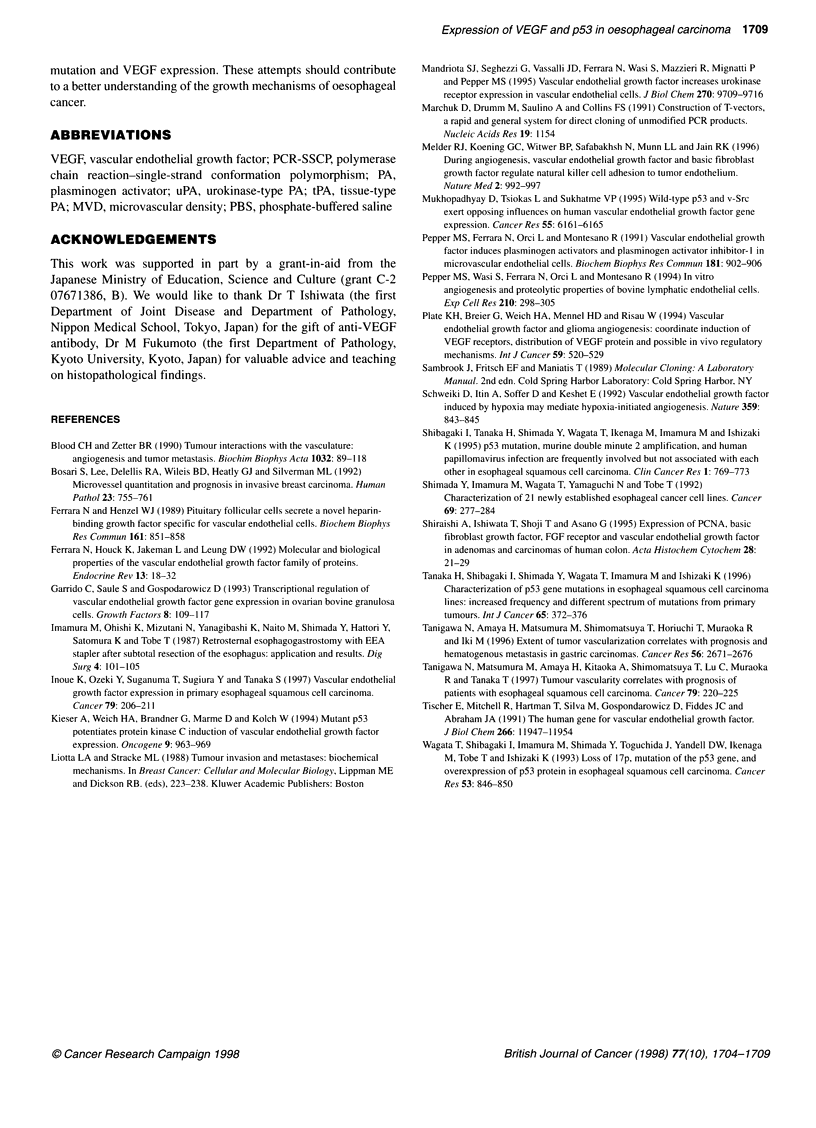# In oesophageal squamous cell carcinoma vascular endothelial growth factor is associated with p53 mutation, advanced stage and poor prognosis.

**DOI:** 10.1038/bjc.1998.281

**Published:** 1998-05

**Authors:** S. Uchida, Y. Shimada, G. Watanabe, H. Tanaka, I. Shibagaki, T. Miyahara, S. Ishigami, M. Imamura

**Affiliations:** Department of Surgery & Surgical Basic Science, Graduate School of Medicine, Kyoto University, Japan.

## Abstract

**Images:**


British Journal of Cancer (1998) 77(10), 1704–1709
© 1998 Cancer Research Campaign

### In oesophageal squamous cell carcinoma vascular endothelial growth factor is associated with p53 mutation, advanced stage and poor prognosis

**S Uchida, Y Shimada, G Watanabe, H Tanaka, I Shibagaki, T Miyahara, S Ishigami, S Arii and M Imamura**

Department of Surgery & Surgical Basic Science, Graduate School of Medicine (formerly First Department of Surgery), Kyoto University, 54-Syogoin kawara-cho, Sakyoku, Kyoto 606-8397, Japan

**Summary** Vascular endothelial growth factor (VEGF) affects malignant tumours by promoting angiogenesis. The tumour-suppressor gene *p53* has been thought to regulate VEGF. We investigated the effect of VEGF on oesophageal carcinoma and the connection between VEGF and p53. One hundred and nine resected oesophageal squamous cell carcinomas were examined. VEGF expression was analysed by immunohistochemical staining. Sixty-five tumours (59.6%, 65 out of 109) were classified as VEGF positive. A significant correlation was found between the VEGF expression and both the depth of invasion ($P = 0.0001$) and lymph node metastasis ($P < 0.0001$). With regard to p53, we compared the expression of VEGF with the mutation of p53, examined using polymerase chain reaction–single-strand conformation polymorphism (PCR-SSCP) and direct sequencing in tumour samples obtained from 36 patients who we have reported previously. The VEGF expression was significantly correlated to p53 mutation ($P = 0.0291$). To evaluate the angiogenesis, microvascular density (MVD) was counted, and endothelial cells were stained immunohistochemically using anti-CD34 monoclonal antibody against 29 cases with invasion limited to the submucosal layer. The average MVD had a tendency to correlate to VEGF expression ($P = 0.1626$). The prognoses of patients with VEGF-positive primary tumours were significantly worse than for those with VEGF-negative primary tumours ($P = 0.0077$). We have assumed that VEGF contributes to aggressive characteristics in oesophageal carcinomas and that VEGF expression might be affected by p53 status.

**Keywords:** vascular endothelial growth factor; p53; oesophageal carcinoma

Oesophageal cancer is one of the most lethal malignant neoplasmas in intestinal diseases. In oesophageal cancer, the penetration of the muscularis mucosa by the primary tumour contributes to an increase in lymphatic metastasis and a worsened prognosis. This is contrary to the case of gastric or colonic carcinomas in which, within the submuscularis layer, lymphatic metastasis is rare and the prognosis is not greatly affected by the penetration of the submuscularis layer.

Carcinoma cells secrete several kinds of cytokines, and their characteristics are influenced by those cytokines. Vascular endothelial growth factor (VEGF) is one of the influential cytokines identified in the media conditioned by bovine pituitary follicular cells (Ferrara et al, 1989). Four types of human VEGF have been identified, including $VEGF_{121}$ (121 amino acids), $VEGF_{165}$ (165 amino acids), $VEGF_{189}$ (189 amino acids) and $VEGF_{206}$ (206 amino acids). All four types of VEGF are secreted in abundance by many kinds of carcinoma cells (Ferrara et al, 1992). Around the carcinoma cells, VEGF is thought to play important roles by directly stimulating endothelial cells to proliferate and migrate, and by activating several proteinase activities that degrade surrounding matrix tissues.

Recent literature about VEGF expression in oesophageal carcinoma describes how VEGF contributes to tumour progression and affects poor prognoses through angiogenesis in oesophageal squamous cell carcinoma (Inoue et al, 1997).

*Received 27 February 1997*
*Revised 4 September 1997*
*Accepted 1 October 1997*

*Correspondence to:* Y Shimada

In contrast, mutations of the tumour-suppressor gene *p53* trigger off tumorigenesis, and interestingly some reports show the possibility that they might be connected with angiogenesis. Mutant p53 could enhance VEGF expression induced by 12-*O*-tetradecanoylphorbol-13-acetate by activating protein kinase C (Kieser et al, 1994). Other possibilities are that wild-type p53 represses VEGF transcription, but p53 mutants don't affect promoter activity, and that wild-type p53 also represses v-Src-mediated VEGF up-regulation (Mukhopadhyay et al, 1995). Although the correlation between VEGF and p53 has been suggested in vitro, there are few reports showing it in clinical materials or in vivo (Plate et al, 1994).

Previously, we detected mutations of p53 in 47% of oesophageal squamous cell carcinoma (Wagata et al, 1993; Shibagaki et al, 1995) and suggested that p53 plays an important role in oesophageal carcinogenesis.

In this study, we first investigated the correlation between the clinicopathological factors of oesophageal carcinoma and the expression of VEGF by immunohistocytochemistry. Secondly, we examined whether VEGF might affect the microvascular increase in oesophageal carcinoma. Finally, we evaluated whether the expression of VEGF correlated to the status of *p53* genes in oesophageal carcinoma.

### MATERIALS AND METHODS

### Clinical materials

Tissues were obtained from oesophageal cancer specimens of 109 patients who underwent oesophagectomies at our institution from

June 1987 to December 1995. The operation techniques used were as previously described (Imamura et al, 1987). The age of the patients ranged from 43 to 84 years; 88 were male, 21 were female (average age, male 64.1 years old, female 61.2 years old). All 109 resected tumours were microscopically examined to identify histological type, extent and mode of cancer invasion, and metastasis to lymph nodes. Histologically, all of the patients had squamous cell carcinoma. Tumour staging was based on the pTNM pathological classification system. They included five patients with stage 0, 30 with stage I, seven with stage IIA, 19 with stage IIB, 30 with stage III and 18 with stage IV (Table 1). Lymph node metastatic lesions of 31 of the 109 patients were also investigated by VEGF staining.

To examine the influence of VEGF on neovascularization, we stained and counted endothelial cells of 29 of the 109 patients whose depths of invasion were the same, i.e. to the submucosal layer.

Specimens were fixed in a 10% formaldehyde solution and embedded in paraffin. Sections, 4 µm thick, were cut and mounted on glass slides.

### Immunohistochemical staining and evaluation

Immunohistochemical staining was performed using the avidin–biotin method. Tissue sections were deparaffinized and rehydrated in water. Endogenous peroxidase was blocked with 0.3% hydrogen peroxide for 30 min. Sections were rehydrated and washed with phosphate-buffered saline (PBS) and incubated with 1.5% normal goat serum in PBS for 30 min at room temperature to block non-specific antibody reaction. Sections were incubated overnight at 4°C with anti-human VEGF polyclonal antibody in PBS containing 1% bovine serum albumin. Anti-human VEGF polyclonal antibody was a kind gift from T Ishiwata of the first Department of Joint Disease and Department of Pathology, Nippon Medical School, Tokyo, Japan (Shiraishi et al, 1995). After six rinses in PBS, sections were incubated for 40 min at room temperature with biotinylated anti-rabbit immunoglobulin G, followed by six washes with PBS, and reacted with an avidin–biotin system, using 0.03% 3,3′-diaminobenzide tetrahydrochloride for about 2 min as chromogen. Sections were counterstained with Mayer's haematoxylin and defined as positive if the membranes or cytoplasm of more than 10% of the tumour cells were stained. Negative controls, prepared by substituting normal rabbit serum for the primary antibody, resulted in no detectable staining.

All slides were analysed by two investigators (SU and IS) without knowledge of the patients' clinical information.

Endothelial cells were stained to examine microvascular density with a monoclonal antibody (QB-END/10; Novocastra Laboratory, Newcastle, UK) against the endothelial marker (CD34) (Tanigawa et al, 1996). Immunohistochemical staining was conducted in the same way as in VEGF. To evaluate microvessel quantitation, slides were scanned at low-power magnification (× 40 to × 100) to identify the areas with the highest number of vessels. In each tumour, the five areas considered to be of the highest density were selected and counted at ×200 power magnification, and the averages were recorded. Vessels with thick muscular walls and vessels of a calibre larger than approximately eight red blood cells were excluded from the count. Single endothelial cells or clusters of endothelial cells, with or without a lumen, were considered to be individual vessels (Bosari et al, 1992).

**Table 1** Characteristics of 109 patients (mean age 64 years, range 43–84 years) and correlation between the expression of VEGF and clinical classification

| | Result of immunostaining No. of patients (%) | | | |
|---|---|---|---|---|
| | VEGF negative | VEGF positive | Total | *P*-value |
| Sex (number of patients) | | | | |
| Male | 36 | 53 | 89 | |
| Female | 8 | 12 | 20 | 0.9704[a] |
| TNM clinical classification | | | | |
| T (primary tumour) | | | | |
| Tis (*n* = 5) | 5 (100) | 0 (0) | 5 | |
| T1 (*n* = 39) | 24 (61.5) | 15 (38.5) | 39 | |
| T2 (*n* = 24) | 5 (20.8) | 19 (79.2) | 24 | |
| T3 (*n* = 29) | 8 (27.6) | 21 (72.4) | 29 | |
| T4 (*n* = 12) | 2 (16.7) | 10 (83.3) | 12 | 0.0001 |
| N (regional lymph nodes) | | | | |
| N0 (*n* = 43) | 30 (69.8) | 13 (30.2) | 43 | |
| N1 (*n* = 66) | 14 (21.2) | 52 (78.8) | 66 | < 0.0001 |
| M (distant metastasis) | | | | |
| M0 (*n* = 91) | 42 (46.2) | 49 (53.8) | 91 | |
| M1 (*n* = 18) | 2 (11.1) | 16 (88.9) | 18 | 0.0073[a] |
| Stage | | | | |
| 0 (*n* = 5) | 5 (100) | 0 (0) | 5 | |
| I (*n* = 30) | 23 (76.7) | 7 (23.3) | 30 | |
| IIA (*n* = 7) | 2 (28.6) | 5 (71.4) | 7 | |
| IIB (*n* = 19) | 4 (21.1) | 15 (78.9) | 19 | |
| III (*n* = 30) | 8 (26.7) | 22 (73.3) | 30 | |
| IV (*n* = 18) | 2 (11.1) | 16 (88.9) | 18 | < 0.0001 |

[a] Fisher's exact test. The TNM clinical classification of the oesophagus, fourth fully revised 1987 edition, is used.

### Oesophageal carcinoma cell lines

To investigate whether oesophageal carcinoma cells express VEGF, we screened 18 oesophageal carcinoma cell lines that had been established in our laboratory (Shimada et al, 1992) We examined their mRNA expression using the Northern hybridization method.

### Preparation of RNA from cell lines

TRIzolTM reagent was purchased from Life Technologies. Cultured cells were lysed with TRIzolTM reagent and scraped with passing cell lysate several times through a 22-gauge needle. The cell lysate was then collected in centrifugation tubes, and the RNA of the cells was extracted according to the product's manual.

### DNA probes for Northern blot analysis

The DNA probe for human $VEGF_{165}$ protein was prepared as follows: 5 µg of human liver total RNA was reverse transcribed with random primers, using a commercial kit (First Strand Synthesis Kit; Pharmacia, Piscataway, NJ, USA). The resulting complementary DNA (cDNA) mixture was subjected to 30 cycles (1 min at 94°C, 1 min at 55° and 1 min at 72°C) of polymerase chain reaction (PCR) amplification using a DNA thermal cycler (Aster, Japan), *Taq* DNA polymerase (Toyobo, Japan) and specific VEGF primers. The following oligonucleotide primers, which were based on the human VEGF cDNA sequence, were used

© Cancer Research Campaign 1998

**Figure 1** Immunohistochemical staining of oesophageal cancer. Specimens were fixed in 10% formaldehyde and embedded in paraffin. An avidin–biotin complex immunoperoxidase method was performed to detect VEGF using anti-VEGF polyclonal antibody. Stained VEGF appears as granules in cytoplasm of carcinoma cells (**A**, magnification ×100; **B**, magnification ×400)

(Tischer et al, 1991): #sense primer, 5′-TTGCTGCTCTACCT-CCAC-3′; and #antisense primer, 5′-AATGCTTTCTCCGCTCTG-3′. Two kinds of PCR products, one of 418 base pairs and the other of 490 base pairs, encoding $VEGF_{165}$ and $VEGF_{189}$, respectively, were obtained. The PCR product that encoded $VEGF_{165}$ was cloned into the *Eco*RV site of the pBluescript SK(–) plasmid (Marchuk et al, 1991), and the inset was confirmed by sequencing. The insert was purified and used as a probe in Northern blot analysis.

### Northern blot analysis

Hybond $N^+$ nylon membrane, rapid hybridization buffer and the Megaprime DNA labelling kit were purchased from Amersham Lifescience (UK). [α-$^{32}$P]dCTP was purchased from ICN Biomedicals (USA). Ten micrograms of mRNA were electrophoresed on a 1.0% agarose gel containing 2.2 M formaldehyde in 1 × 3-(*N*-morpholino)propanesulphonic acid (MOPS) buffer (20 mM MOPS, 5 mM sodium acetate, 1 mM EDTA-2Na) and then transferred to a Hybond $N^+$ nylon membrane by capillary blotting, followed by ultraviolet (UV) cross-linkage.

Blotted membranes were prehybridized in rapid hybridization buffer at 65°C for 30 min. [α-$^{32}$P]dCTP-labelled human $VEGF_{165}$ cDNA was prepared using a Megaprime DNA labelling kit. Sephadex-G column was used to remove the free radioisotope. Hybridization was performed at 65°C for 2 h and then the membranes were washed twice for 10 min at room temperature in 2 × standard saline citrate (SSC) (300 mM sodium chloride, 30 mM sodium citrate) with 0.1% sodium dodecyl sulphate (SDS). If background radioactivity was high, membranes were washed again at 65°C for 20 min in 1 × SSC with 0.1% SDS, and then at 65°C for 15 min in 0.1 × SSC with 0.1% SDS. Membranes were exposed to Fuji radiographic films at –80°C for appropriate intervals. All techniques were performed according to standard methods described previously (Sambrook et al, 1989). Expression of mRNA was quantified using the BAS station system (Fuji photo film, Japan).

### Statistical analysis

Results among VEGF, p53 mutation and clinical factors were analysed using $\chi^2$ analysis and Fisher's exact test. Microvascular count was analysed using an unpaired *t*-test. Survival curves of the patients were calculated using the Kaplan–Meier method and statistically analysed using the generalized Wilcoxon test. Multivariate analysis was performed using the Cox proportional hazards model.

**Table 2** The stained VEGF detection between primary tumours and lymph node metastatic lesions

| | No. of patients with lymph node metastatic lesions | | |
|---|---|---|---|
| **Primary tumour** | **VEGF negative** | **VEGF positive** | ***P*-value** |
| VEGF negative | 3 | 2 | |
| VEGF positive | 11 | 15 | 0.4033[a] |

[a]Fisher's exact test.

### RESULTS

VEGF was mainly detected on the cytoplasms or the membranes of the carcinoma cells (Figure 1). Carcinoma cells seemed to stain stronger on the marginal region of the cancer nest than in the tumour centre. Few cancer nests were stained diffusely. Vascular endothelial cells beside strongly stained cancer nests were also stained. Cancer cells stained more intensely than smooth muscle. Overall, 65 (59.6%) tumours were classified as VEGF positive.

Table 1 shows the correlation between VEGF expression and various factors. The VEGF-positive rate exceeded its negative rate when the tumours grew deeper than T2 level. the VEGF expression also correlated to lymph nodal metastasis. To evaluate the status of VEGF at metastatic lymph nodes, lymph node metastatic lesions were stained immunochemically with anti-VEGF antibody. Interestingly, we could not find a relationship between the result of primary lesions and that of lymph node metastatic lesions (Table 2). Of the 109 patients, a small group had distant metastasis. When distant metastasis was detected, a tendency for a higher proportion of VEGF-positive than VEGF-negative cases was found. As a result, the VEGF-positive rate became significantly higher ($P<0.0001$) with stage advance.

To evaluate the vascular sprouting, the number of endothelial cells stained by anti-CD34 monoclonal antibody was counted

© Cancer Research Campaign 1998

**Figure 2** Immunohistochemical staining for endothelial cells in a specimen of oesophageal carcinoma. Anti-CD34 monoclonal antibody was used to detect endothelial cells. Endothelial cells are stained as vascular ring or clusters near the tumour (magnification ×200)

**Figure 3** Association between microvessel count and the expression of VEGF. The small dots show the averages of microvessel count out of five microscopic 200 × fields. The parallel bar and large dots show standard deviation and the mean value

(Figure 2). Eleven patients out of 29 immunohistochemically stained cases were defined as VEGF positive. The average of microvascular density (MVD) in VEGF-positive patients was higher than that of VEGF-negative patients, but we could not find a significant difference between them ($P = 0.1626$) (Figure 3).

With regards to the 36 patients whom we had previously screened with p53 mutations using polymerase chain reaction–single-strand conformation polymorphism (PCR-SSCP) and direct sequencing (Shibagaki et al, 1995), we identified p53 mutations in 16 (44.4%) of 36 tumours in which we found mutations in exons 4–10. Twenty-two tumours (61.1%) were classified as VEGF positive. We found that the number of VEGF-positive tumours in p53-mutant tumours

**Table 3** The association between VEGF and p53

| | Result of immunostaining No. of patients | | |
|---|---|---|---|
| *p53* gene status | VEGF negative | VEGF positive | *P*-value |
| Mutant type | 3 | 13 | |
| Wild type | 11 | 9 | 0.0291[a] |

[a]Fisher's exact test. p53 status was investigated by PCR-SSCP and direct sequencing.

**Figure 4** The relation between the influence of VEGF and survival rate. Survival curves of the patients were calculated using the Kaplan–Meier method. Seven out of 109 patients were non-curative cases, therefore we excluded these patients in this analysis was significantly higher than in the wild-type-p53 tumours (Table 3) ($P = 0.0291$).

The survival rates of 102 patients who received curative resection (R0) were examined. (Fifteen out of 18 stage IV patients did not have organ metastases, except for cervical lymph nodal metastases, which were resected.) Naturally, the survival rate of VEGF-positive cases was significantly lower than that of VEGF-negative cases, proportional to the clinical classification ($P = 0.0077$) (Figure 4). Multivariate analysis using Cox's proportional hazards model revealed that there was no significant correlation between the expression of VEGF and the patients' outcome (Table 4).

In our 18 oesophageal carcinoma cell lines, we found various degrees of VEGF mRNA expression among all 18 cell lines. The ratio of VEGF mRNA expression to G3PDH varied from 0.547 to 6.45, but no correlation was found between the expression of VEGF and p53 mutations (Tanaka et al, 1996). Figure 5 shows representative results of the mRNA of VEGF.

### DISCUSSION

Our data show a drastic increase in the number of patients whose primary tumours were defined as VEGF positive between T1 and T2 in the depth of invasion [TNM classification of the oesophagus: tumour invades lamina propria or submucosa, (T1); tumour invades muscularis propria, (T2)]. The number of cases involving lymph node metastasis increased when the tumour invaded into the submucosal layer. These increases may correlate with each

© Cancer Research Campaign 1998

**Table 4** Value of five variables in predicting overall survival of 102 patients

| Factors | Estimate | Risk ratio | 95% Confidence interval | Chi square | *P*-value |
|---|---|---|---|---|---|
| pT | 0.765 | 2.148 | 1.359–3.491 | 11.120 | 0.0009 |
| pN | 0.045 | 1.046 | 0.264–4.356 | 0.004 | 0.9493 |
| Sex | 1.437 | 4.210 | 1.471–17.794 | 7.896 | 0.0050 |
| Age | 1.013 | 1.013 | 0.975–1.053 | 0.456 | 0.4994 |
| VEGF | 1.804 | 1.804 | 0.750–5.011 | 1.160 | 0.1976 |

**Figure 5** Northern blot analysis of VEGF expression and p53 status in representative oesophageal cancer cell lines. G3PDH, control of RNA loading by hybridization with a G3PDH probe. Mutations of p53 were found in all these cell lines other, and suggest that VEGF may influence both the growth of the oesophageal carcinoma in transmural invasion and the lymph node metastasis by affecting at primary sites.

Invasion into the lymphatics could occur through several routes: by direct invasion of the tumour front into lymph nodes; by invasion of individual cells extruded from the tumour interstitium into the surrounding tissue; or by the passage of tumour cells from the bloodstream into the lymphatics via the lymphatic–haematogenous communications (Liotta et al, 1988). Tumour angiogenesis induced by VEGF may facilitate this process by increasing tumour volume and superficial area, thus enhancing tumour cell–lymphatic contact at the growing margin of the tumour. It may also increase the numbers of lymphatic–haematogenous communications, allowing more intravascular micrometastasis to enter the lymphatic spaces (Blood et al, 1990). VEGF is also known as vascular permeability factor and is thought to activate natural killer cells into connecting with endothelial cells through adhesional molecules (Melder et al, 1996). Furthermore, VEGF has been shown to induce urokinase-type plasminogen activator (uPA), tissue-type PA (tPA) and plasminogen activator inhibitor-1 (PAI-1) in vascular endothelial cells and bovine lymphatic endothelial cells (Pepper et al, 1991, 1994). VEGF also induces uPA receptor in vascular endothelial cells (Mandriota et al, 1995). VEGF might facilitate lymph nodal metastasis by degrading tumour marginal extracellular matrix through uPA and the uPA receptor system as well as through matrix metalloproteinases (MMP), some of which might be activated by plasmin. These connections may facilitate the extravasation of the carcinoma cells at primary sites and may be one cause of VEGF-positive staining of primary tumours increasing lymph node metastasis. In contrast, our data showed that the expression of VEGF at metastatic lymph nodes did not depend on the expression of VEGF at primary tumours.

With regard to the regulation against the expression of VEGF, several factors, such as hypoxia, oestrogen levels and p53 mutation, were reported to induce the expression of VEGF (Schweiki et al, 1992; Garrido et al, 1993; Kieser et al, 1994; Mukhopadhyay et al, 1995). Our results show that the mutation of p53 coexisted with the expression of VEGF in oesophageal carcinoma patients, but the detailed mechanisms determining how mutant p53 induces VEGF expression are not known.

It has been reported that increase of the microvessels around the tumour might worsen the prognosis of oesophageal carcinoma. (Tanigawa et al, 1997). Inoue et al (1997) reported from their immunohistochemical study of vessel density using anti-factor VIII antibody that VEGF might mediate angiogenesis in oesophageal carcinoma. To investigate whether VEGF really induces microvessels around the oesophageal tumour, we examined the microvascular density by staining endothelial cells immunohistochemically, using anti-CD34 antibody. Anti-CD34 antibody is more specific than anti-factor VIII antibody and, because the average vessel counts were approximately three times higher with CD34 staining than with factor VIII staining (Tanigawa et al, 1997), it may be controversial to evaluate vessel density using anti-factor VIII antibody. Although we could not find a significant difference in microvascular density between VEGF-positive cases and VEGF-negative cases, we found that the VEGF-positive cases tended to show a higher number of microvascular counts than VEGF-negative cases. Even if they were normal tissues, the basic number of the endothelial cells in each layer varied. In the case of carcinoma tissues, the endothelial cell count at different layers may be influenced by the variety of the basic number of them. In many cases, the structure of the muscular layer tissues invaded by the carcinoma was destroyed too severely to investigate endothelial cell numbers, so our research was limited to 29 cases whose depths of invasion were at the submucosal layer. We could not show a significant difference in endothelial cell numbers between VEGF-positive cases and VEGF-negative cases.

Finally, although there is the possibility that cancer cell lines may change their natural character, we confirmed VEGF expression in oesophageal cancer by detecting its mRNA in cell lines. Our results may indicate that the expression of VEGF is common in oesophageal cancer and well correlated with p53 status in vivo; however the same correlation was not shown in vitro. There may be the involvement of other factors. We have already examined the p53 status of these cell lines (Tanaka et al, 1995), so we are now analysing them for direct evidence of a correlation between p53

© Cancer Research Campaign 1998

mutation and VEGF expression. These attempts should contribute to a better understanding of the growth mechanisms of oesophageal cancer.

### ABBREVIATIONS

VEGF, vascular endothelial growth factor; PCR-SSCP, polymerase chain reaction–single-strand conformation polymorphism; PA, plasminogen activator; uPA, urokinase-type PA; tPA, tissue-type PA; MVD, microvascular density; PBS, phosphate-buffered saline

### ACKNOWLEDGEMENTS

This work was supported in part by a grant-in-aid from the Japanese Ministry of Education, Science and Culture (grant C-2 07671386, B). We would like to thank Dr T Ishiwata (the first Department of Joint Disease and Department of Pathology, Nippon Medical School, Tokyo, Japan) for the gift of anti-VEGF antibody, Dr M Fukumoto (the first Department of Pathology, Kyoto University, Kyoto, Japan) for valuable advice and teaching on histopathological findings.

### REFERENCES

Blood CH and Zetter BR (1990) Tumour interactions with the vasculature: angiogenesis and tumor metastasis. *Biochim Biophys Acta* **1032**: 89–118

Bosari S, Lee, Delellis RA, Wileis BD, Heatly GJ and Silverman ML (1992) Microvessel quantitation and prognosis in invasive breast carcinoma. *Human Pathol* **23**: 755–761

Ferrara N and Henzel WJ (1989) Pituitary follicular cells secrete a novel heparin-binding growth factor specific for vascular endothelial cells. *Biochem Biophys Res Commun* **161**: 851–858

Ferrara N, Houck K, Jakeman L and Leung DW (1992) Molecular and biological properties of the vascular endothelial growth factor family of proteins. *Endocrine Rev* **13**: 18–32

Garrido C, Saule S and Gospodarowicz D (1993) Transcriptional regulation of vascular endothelial growth factor gene expression in ovarian bovine granulosa cells. *Growth Factors* **8**: 109–117

Imamura M, Ohishi K, Mizutani N, Yanagibashi K, Naito M, Shimada Y, Hattori Y, Satomura K and Tobe T (1987) Retrosternal esophagogastrostomy with EEA stapler after subtotal resection of the esophagus: application and results. *Dig Surg* **4**: 101–105

Inoue K, Ozeki Y, Suganuma T, Sugiura Y and Tanaka S (1997) Vascular endothelial growth factor expression in primary esophageal squamous cell carcinoma. *Cancer* **79**: 206–211

Kieser A, Weich HA, Brandner G, Marme D and Kolch W (1994) Mutant p53 potentiates protein kinase C induction of vascular endothelial growth factor expression. *Oncogene* **9**: 963–969

Liotta LA and Stracke ML (1988) Tumour invasion and metastases: biochemical mechanisms. In *Breast Cancer: Cellular and Molecular Biology*, Lippman ME and Dickson RB. (eds), 223–238. Kluwer Academic Publishers: Boston

Mandriota SJ, Seghezzi G, Vassalli JD, Ferrara N, Wasi S, Mazzieri R, Mignatti P and Pepper MS (1995) Vascular endothelial growth factor increases urokinase receptor expression in vascular endothelial cells. *J Biol Chem* **270**: 9709–9716

Marchuk D, Drumm M, Saulino A and Collins FS (1991) Construction of T-vectors, a rapid and general system for direct cloning of unmodified PCR products. *Nucleic Acids Res* **19**: 1154

Melder RJ, Koening GC, Witwer BP, Safabakhsh N, Munn LL and Jain RK (1996) During angiogenesis, vascular endothelial growth factor and basic fibroblast growth factor regulate natural killer cell adhesion to tumor endothelium. *Nature Med* **2**: 992–997

Mukhopadhyay D, Tsiokas L and Sukhatme VP (1995) Wild-type p53 and v-Src exert opposing influences on human vascular endothelial growth factor gene expression. *Cancer Res* **55**: 6161–6165

Pepper MS, Ferrara N, Orci L and Montesano R (1991) Vascular endothelial growth factor induces plasminogen activators and plasminogen activator inhibitor-1 in microvascular endothelial cells. *Biochem Biophys Res Commun* **181**: 902–906

Pepper MS, Wasi S, Ferrara N, Orci L and Montesano R (1994) In vitro angiogenesis and proteolytic properties of bovine lymphatic endothelial cells. *Exp Cell Res* **210**: 298–305

Plate KH, Breier G, Weich HA, Mennel HD and Risau W (1994) Vascular endothelial growth factor and glioma angiogenesis: coordinate induction of VEGF receptors, distribution of VEGF protein and possible in vivo regulatory mechanisms. *Int J Cancer* **59**: 520–529

Sambrook J, Fritsch EF and Maniatis T (1989) *Molecular Cloning: A Laboratory Manual*. 2nd edn. Cold Spring Harbor Laboratory: Cold Spring Harbor, NY

Schweiki D, Itin A, Soffer D and Keshet E (1992) Vascular endothelial growth factor induced by hypoxia may mediate hypoxia-initiated angiogenesis. *Nature* **359**: 843–845

Shibagaki I, Tanaka H, Shimada Y, Wagata T, Ikenaga M, Imamura M and Ishizaki K (1995) p53 mutation, murine double minute 2 amplification, and human papillomavirus infection are frequently involved but not associated with each other in esophageal squamous cell carcinoma. *Clin Cancer Res* **1**: 769–773

Shimada Y, Imamura M, Wagata T, Yamaguchi N and Tobe T (1992) Characterization of 21 newly established esophageal cancer cell lines. *Cancer* **69**: 277–284

Shiraishi A, Ishiwata T, Shoji T and Asano G (1995) Expression of PCNA, basic fibroblast growth factor, FGF receptor and vascular endothelial growth factor in adenomas and carcinomas of human colon. *Acta Histochem Cytochem* **28**: 21–29

Tanaka H, Shibagaki I, Shimada Y, Wagata T, Imamura M and Ishizaki K (1996) Characterization of p53 gene mutations in esophageal squamous cell carcinoma lines: increased frequency and different spectrum of mutations from primary tumours. *Int J Cancer* **65**: 372–376

Tanigawa N, Amaya H, Matsumura M, Shimomatsuya T, Horiuchi T, Muraoka R and Iki M (1996) Extent of tumor vascularization correlates with prognosis and hematogenous metastasis in gastric carcinomas. *Cancer Res* **56**: 2671–2676

Tanigawa N, Matsumura M, Amaya H, Kitaoka A, Shimomatsuya T, Lu C, Muraoka R and Tanaka T (1997) Tumour vascularity correlates with prognosis of patients with esophageal squamous cell carcinoma. *Cancer* **79**: 220–225

Tischer E, Mitchell R, Hartman T, Silva M, Gospondarowicz D, Fiddes JC and Abraham JA (1991) The human gene for vascular endothelial growth factor. *J Biol Chem* **266**: 11947–11954

Wagata T, Shibagaki I, Imamura M, Shimada Y, Toguchida J, Yandell DW, Ikenaga M, Tobe T and Ishizaki K (1993) Loss of 17p, mutation of the p53 gene, and overexpression of p53 protein in esophageal squamous cell carcinoma. *Cancer Res* **53**: 846–850

© Cancer Research Campaign 1998